# Glucose-Coated Gold Nanoparticles Transfer across Human Brain Endothelium and Enter Astrocytes *In Vitro*

**DOI:** 10.1371/journal.pone.0081043

**Published:** 2013-12-05

**Authors:** Radka Gromnicova, Heather A. Davies, Peddagangannagari Sreekanthreddy, Ignacio A. Romero, Torben Lund, Ivan M. Roitt, James B. Phillips, David K. Male

**Affiliations:** 1 Biomedical Research Network, The Open University, Milton Keynes, United Kingdom; 2 Department of Natural Sciences, Middlesex University, London, United Kingdom; Okayama University, Japan

## Abstract

The blood-brain barrier prevents the entry of many therapeutic agents into the brain. Various nanocarriers have been developed to help agents to cross this barrier, but they all have limitations, with regard to tissue-selectivity and their ability to cross the endothelium. This study investigated the potential for 4 nm coated gold nanoparticles to act as selective carriers across human brain endothelium and subsequently to enter astrocytes. The transfer rate of glucose-coated gold nanoparticles across primary human brain endothelium was at least three times faster than across non-brain endothelia. Movement of these nanoparticles occurred across the apical and basal plasma membranes via the cytosol with relatively little vesicular or paracellular migration; antibiotics that interfere with vesicular transport did not block migration. The transfer rate was also dependent on the surface coating of the nanoparticle and incubation temperature. Using a novel 3-dimensional co-culture system, which includes primary human astrocytes and a brain endothelial cell line hCMEC/D3, we demonstrated that the glucose-coated nanoparticles traverse the endothelium, move through the extracellular matrix and localize in astrocytes. The movement of the nanoparticles through the matrix was >10 µm/hour and they appeared in the nuclei of the astrocytes in considerable numbers. These nanoparticles have the correct properties for efficient and selective carriers of therapeutic agents across the blood-brain barrier.

## Introduction

A major challenge for the pharmaceutical industry is the delivery of therapeutic biomolecules and transgenes into the central nervous system (CNS). The blood-brain barrier (BBB), formed by microvascular endothelium, pericytes and astrocytes, prevents the movement of most larger hydrophilic molecules (>1 kDa) and many toxic agents. The key elements of the barrier are continuous tight-junctions between endothelial cells, which prevent molecules from diffusing into the brain by the paracellular route, and ABC-transporters that actively pump xenobiotics out of the brain [1], [2]. In addition, brain endothelial cells have only low levels of pinocytotic activity [3]. As a result, many drugs and larger biomolecules, including cytokines and gene-modifying therapies, which have considerable potential for the treatment of CNS disease, are excluded by the endothelial barrier [4]–[7].

Considerable efforts have been made to find a way of overcoming the blood-brain barrier, including the use of nanoparticles as carriers [8]. Gold nanoparticles have the advantage of easy production and chemical stability and they have recently been tested in nanomedicine for both diagnosis and therapy [9]. The gold core is inert but it does interact with biological material and can have biological effects. To address this, a variety of sizes and surface modifications have been investigated which affect the specific behaviour of the nanoparticles [10]–[12]. However, there is comparatively little data on which nanoparticles are selective for endothelium from different tissues.

Nanoparticle transport into a cell depends highly on the size and surface coating of the nanoparticles. Relatively small gold nanoparticles (<50 nm) may enter cells via an endocytic pathway [13], [14] and it has been calculated that a size of 27–30 nm is optimal for endocytosis [15]. It has been thought that gold nanoparticles do not enter the nucleus unless the cell is apoptotic [16]. In contrast, they are often trapped in vesicles (endosomes) [17]–[20] and can end up in lysosomes, with sensitive cargo being digested by lysosomal enzymes, which presents an obstacle for drug/gene delivery into tissues. Hence, in relation to the blood-brain barrier, the ideal components of a CNS nanoparticle-based drug delivery system are:

1. movement through the cellular cytosol, 2. selectivity for the brain endothelium, 3. the ability to cross the brain endothelium intact, 4. uptake by the target cell within the CNS and 5. low toxicity and immunogenicity.

How can selectivity for the CNS be achieved? Since brain endothelium has a number of specific receptors and transporters which allow influx of nutrients into the brain, their ligands have been exploited in attempts to develop CNS specific nanoparticles [21]. For example, nanoparticles coated with ApoE (targeting the LDL receptor) or OX26 antibody (targeting the transferrin receptor) have both been used in CNS drug delivery [17], [21]. An alternative approach relies on the physical properties of the nanoparticles; it has been found that small gold nanoparticles can directly penetrate the plasma membrane, and this property also depends on the surface coating and structure of the nanoparticle [22], [23]. Moreover, the biophysical surface properties of brain endothelium are different from non-brain endothelium with a high negative surface charge, due to sulphated proteoglycans [24]. The distinctive properties of brain endothelium imply that selective targeting of nanoparticles to the CNS is possible.

In this study, we have chosen glucose-coated gold nanoparticles, 4 nm in size, with a 2 nm gold core [25]. These nanoparticles are considerably smaller than nanoparticles used in related studies [17]. Glucose-coated nanoparticles were initially selected because the glucose transporter Glut-1 is expressed on brain endothelium and astrocytes. However, the experimental data indicated that it is the biophysical properties of these nanoparticles rather than receptor-binding which is important for their transfer across brain endothelium.

We tested whether these nanoparticles can be used as a potential carrier across the blood-brain barrier, focusing on (1) studying localization inside the cell; (2) comparison of uptake of these nanoparticles by brain endothelium compared with endothelia from other tissues (bone marrow and coronary artery) in order to establish whether the glucose-coated nanoparticles are CNS-selective; and (3) studying transfer across the brain endothelium and into astrocytes using an *in vitro* 3D co-culture model.

We have also developed a novel model of the blood brain barrier, in which human astrocytes are cultured in a 3-dimensional (3D) collagen gel, beneath a monolayer of human brain endothelium. This model is based on a 3D rat glial cell culture system previously developed in our laboratories [26], [27], which has been modified to include primary human astrocytes and the brain endothelial cell line hCMEC/D3 [28]. To investigate the distribution of gold nanoparticles in cells, we have used transmission electron microscopy (TEM) to give quantitative data on the localization of the nanoparticles in different subcellular compartments.

## Materials and Methods

### Ethical Statement

Anonymous tissue donations from elective surgical resections were made according to a protocol approved by Oxfordshire REC-C (07/H0606/97).

### Endothelial, Astrocyte and Fibroblast Cultures

Primary human brain microvessel endothelium (1-BEC) was obtained from surgical resection, undertaken to treat epilepsy, with the informed, written consent of the patient. The cells were isolated from a small area of unaffected tissue at the tip of the temporal lobe, by collagenase/dispase digestion and isolation on BSA and percoll gradients as previously described [29]. The cells were cultured (passage-1) on collagen-coated flasks or tissue culture inserts in EBM-2 MV medium (Lonza, Basel, Switzerland) supplemented with 10% foetal bovine serum, hydrocortisone, VEGF, epidermal growth factor (EGF), insulin-like growth factor I (IGF-I), human fibroblast growth factor (FGF), ascorbic acid, amphotericin-B and gentamicin sulphate according to the manufacturer’s formulation. This same medium and conditions were used for culturing human fibroblasts.

The human cerebral microvessel endothelial cell line hCMEC/D3 [28] at passage 24–30 and primary human coronary artery endothelial cells (CoAEC, Lonza; Cat. No. CC*-*2585) were cultured in EBM-2 medium as described above but with 2.5% foetal bovine serum. The human bone marrow endothelial cell line BMEC [30] (kindly supplied by Dr Babette Weksler, Cornell, University) was cultured in DMEM (Sigma-Aldrich) supplemented with 10% foetal bovine serum with 100 U/ml penicillin and 100 µg/ml streptomycin (Invitrogen, UK). All the endothelial cells were cultured at 37°C in a humidified atmosphere containing 5% CO_2_, unless otherwise indicated.

Human foetal cortical astrocytes (used at passage 3–6), were obtained from ScienCell Research Laboratories (Carlsbad, Ca). The cells were maintained on collagen type-I coated tissue culture dishes in human astrocyte medium (ScienCell, Carlsbad, Ca) including 2% foetal bovine serum and recommended growth supplements.

### 3D Collagen Gel Astrocyte Cultures and Astrocyte/Endothelial Co-cultures

Collagen gels containing 1.2×10^6^ astrocytes per ml were prepared in 24-well plates, with an initial volume of 450 µl cellular collagen gel per well. Gels were composed of a 10% cell suspension of human astrocytes (in DMEM), 10% 10x minimum essential medium (MEM; Sigma-Aldrich) and 80% type I rat tail collagen (2.5 mg/ml; First Link, Wolverhampton, UK([31]. The collagen was diluted from a 5 mg/ml 0.6% acetic acid stock using water, then mixed with MEM and neutralised using sodium hydroxide (assessed by colour change of the phenol red indicator), then the mixture was added to the cell suspension and mixed to ensure even distribution of cells before transfer to the pre-warmed 24-well plate. Gelation took ∼10 min at 37°C. The gels were overlaid with astrocyte medium and incubated for 2 hrs before being stabilised using RAFT™ absorbers (TAP Biosystems, Royston, UK) for 15 min to remove fluid and reduce gels to approximately 10% of their original volume. Astrocyte gels were incubated for a further 24 hrs in astrocyte medium before being overlaid with hCMEC/D3 cells at a density of 50,000 cells/cm^2^.

These co-cultures were incubated for 3 days in EBM2 medium with 2.5% FBS before the nanoparticles were applied to the apical surface in fresh media for 1, 3 or 8 hrs. After incubation with nanoparticles, co-culture gels were washed ×3 in PBS and fixed in 2.5% glutaraldehyde in 0.1 M Sörensons phosphate buffer for at least 1 hour. They were further processed for transmission electron microscopy (TEM) as described below for transwell inserts.

### Gold Nanoparticle Transport Assay

Gold nanoparticles were synthesised by Midatech Ltd (Abingdon, UK) as described previously [25] using a modification of the Brust-Schiffrin method [32], replacing the 2-phase synthesis with a single phase (water), as the ligands are water-soluble. The gold core (diameter ∼2 nm) was covalently coated with either β2- mercaptoethoxy-glucose or glutathione, producing nanoparticles coated with either glucose or glutathione, which increased the hydrodynamic diameter of the particle to approximately 4 nm. The nanoparticles have a structured surface with bands of ligand as previously described for other nanoparticles of this class [22], [33]. The particle size was checked by TEM and the chemical characterisation was carried out by Malvern Instruments Ltd. (Malvern, UK). The glucose-coated nanoparticles have a mean molecular mass of 27 kDa.

For transfer assays, 12-well transwell inserts (Corning Costar) were coated with collagen and seeded with 40,000 cells per well and incubated for 2 or 3 days to reach confluence. The cells were then washed in HBSS and gold nanoparticles were added to the fresh culture medium (0.5 ml) in the upper chamber to a final concentration of 8.16 µg/ml. The cultures were then incubated for 0 hrs (10 min) to 22 hrs at 37°C. After the incubation with nanoparticles, the inserts were washed ×3 in PBS and fixed in 2.5% glutaraldehyde in 0.1 M Sörensons phosphate buffer (PB) for 1 hour at room temperature. They were further processed for TEM, as described below for inserts.

In experiments where inhibitor treatments were used, the antibiotics were present for 1 hour before the experiment and throughout the migration assay (3 hrs). The drugs selected were cytochalasin-B, cytochalasin-D, nocodazole, nystatin and chlorpromazine (Sigma-Aldrich). The concentrations were selected for their ability to block vesicular transport in human brain endothelial cells, and lack of cytotoxicity [34]–[37]. We also confirmed that the cells were not affected by the agents at the given concentrations for at least 8 hrs, as assessed by light and electron microscopy.

### Transmission Electron Microscopy (TEM)

Gold nanoparticles were visualized by silver enhancement (Aurion, Netherlands) for 45 min at room temperature. Post-fixation was carried out with 1% (w/v) osmium tetroxide in 0.1 M PB for 1 hour and the transwell inserts were then washed in 0.1 M PB for 10 min. The polyester membrane with the cultures were excised from the insert and randomly cut into 2 segments of 3–5 mm×2 mm. These segments were progressively dehydrated in a graded series of ethanol from 30% to 100%, embedded in Epon resin and polymerised at 60°C for 48 hrs. Ultrathin sectioning was performed using a Diatome diamond knife producing sections of 80–90 nm thickness, which were then collected on 2×1 mm copper grids with pioloform film. The sections were counterstained at room temperature with 4% aqueous uranyl acetate for 35 min, washed three times, immersed in Reynolds lead citrate for 10 min and finally washed three times before air-drying. The sections were observed on a transmission electron microscope JEM-1400 operated at an accelerating voltage of 80 kV using a magnification of ×5000 up to ×25,000.

To test if silver enhancement of cultures gives any background labelling, we used cultures that did not contain any nanoparticles (negative control) which were processed and treated as cultures containing gold nanoparticles (above).

### Sampling and Analysis of TEM Data

To choose representative data, a systematic sampling method was used. Twenty-five images were taken from each section at regular intervals, i.e. every fourth microscopic field containing a cell. After this, every picture was analysed separately by counting the observed nanoparticles which were assigned into six categories (Table 1). The length of the cell membrane visible in each picture (apical or basal membrane) was measured using software Image-J version 1.43. Data points are based on a measurement of at least 50 cells from each experimental treatment or time-point (2 technical replicates with 25 images per replicate), (**Fig. S1**). Each experiment was performed 2–4 times and the figures show data from a representative experiment. The data are expressed either as nanoparticles per micron of plasma membrane or nanoparticles per cell. Note that the figures on the graphs refer to an 85 nm thick section of the cell, and estimates of the total number of nanoparticles per cell are made by a calculation based on the area of the monolayers and the numbers of cells.

**Table 1:** The categories that were established to sort localization of glucose-coated gold nanoparticles in cells.

Category	Description of nanoparticles (NPs) belonging to this category
Upper membrane	NPs adhered to the apical surface of cell membrane
Lower membrane	NPs adhered to the basal extracellular surface of the plasma membrane of a cell that was attached to the transwell insert; NPs accumulated between the polyester membrane of the insert and the lower plasma membrane of the cells.
Cytosol	NPs freely distributed in cytosol, usually not clumped
Vesicles	NPs located in endosomes, lysosomes, granules, vacuoles or mitochondria*
Junction	NPs in intercellular junctions
Nucleus	NPs inside the nucleus

* even though nanoparticles were not definitely observed in mitochondria, we cannot exclude them from this category as during sectioning it is not always possible to unambiguously identify every membrane surrounded organelle or granule.

To evaluate astrocytes in 3-dimensional collagen gels, images were taken of all astrocytes in each section; the area of each cell and nucleus was measured (in microns squared) using Image-J and the nanoparticles counted and assigned to the categories listed in Table 1.

For astrocytes in co-culture with hCMEC/D3 cells in 3-D gels, at least 240 astrocytes were evaluated in each gel, in order to identify 50 cells containing nanoparticles in a gel (data collected from 1 to 3 different ultrathin sections from each gel). The distance of each astrocyte from the basal plasma membrane of the endothelium was also measured. All treatments were performed in duplicate and the experiment was performed twice, with representative data shown.

### Viability Assay

An MTT assay was performed in a 96-well plate format to assess cytotoxicity of the glucose-coated gold nanoparticles on hCMEC/D3 cells. The cells were cultured for 2 days (seeding density 20,000 cells per well) in EBM-2 medium. They were washed and medium containing gold nanoparticles with different concentrations (4, 8, 16 and 32 µg/ml) was added. All treatments were performed in quadruplicate. The cells were incubated for 24 hrs. Negative controls were cells without nanoparticles; positive controls were cells treated with digitonin (30 µg/ml for 30 min). Wells containing medium only were used as a blank. After the incubation, the medium was removed from all wells and medium with 0.5 mg/ml MTT (3-(4,5-Dimethylthiazol-2-yl)-2,5-diphenyltetrazolium bromide; Sigma-Aldrich) was added to each well. The plate was incubated for 3.5–4 hrs, the solution carefully aspirated from the wells and 100 µl of DMSO (Sigma-Aldrich) was added to each well. The plate was placed on an orbital shaker for 15 min and absorbance was read at 540 nm on a plate reader.

### Statistical Analysis

Comparison of different treatments was initially carried out by one way Anova. If significant differences were found (p<0.05), then the data was either analysed by Tukey’s test for pairwise comparisons or Dunnett’s multiple comparison test to compare different treatments with the control. The analysis was carried out using Prism ‘Graphpad’ software.

## Results

### Cellular Localization of Glucose-coated Gold Nanoparticles

To determine whether glucose-coated gold nanoparticles can cross human brain endothelium, we applied the nanoparticles to the apical surface of endothelial cell monolayers, incubated the cultures for 0–22 hrs and detected them by transmission electron microscopy (TEM). We used silver enhancement to increase the size of nanoparticles to observable size (∼20 nm). This works on a principle of deposition of silver on the nanoparticle surface. We confirmed that the silver enhancement itself does not cause background labelling on cultures without nanoparticles (data not shown).

**Figure 1:**
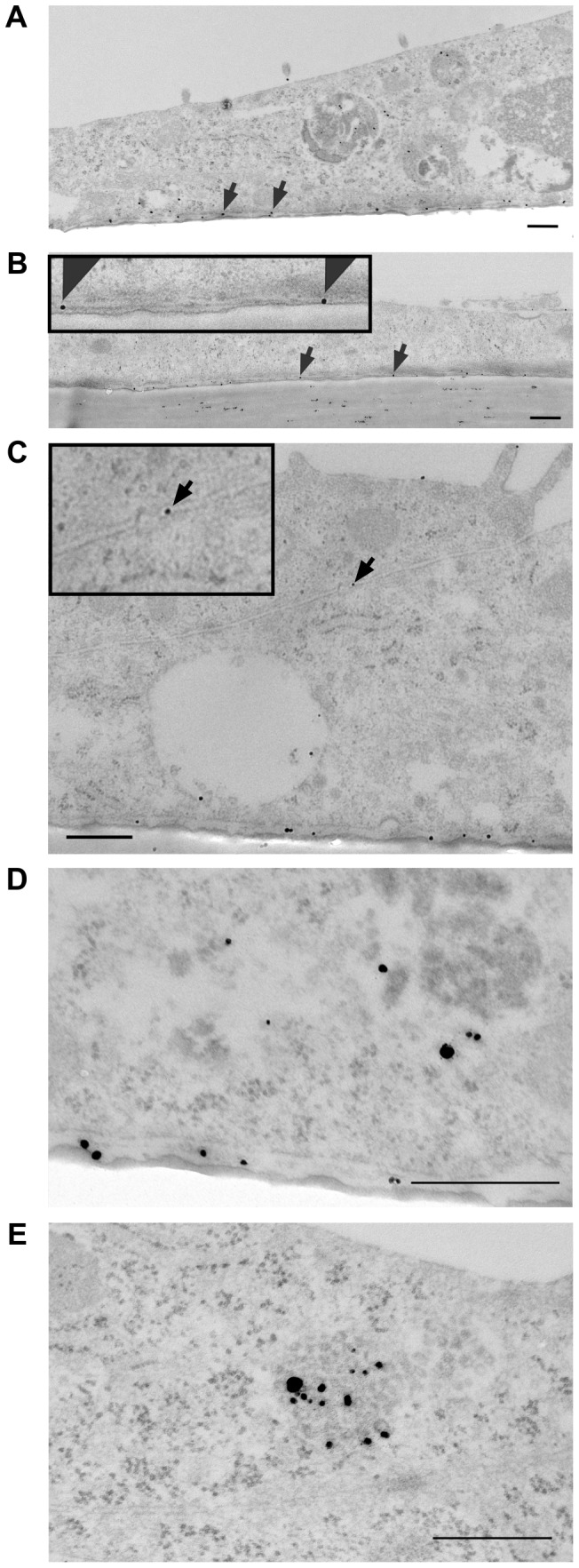
Electron micrographs of brain endothelial cells with glucose-coated gold nanoparticles. (a) hCMEC/D3 cells and (b) primary human brain endothelium 8 hours after application of glucose-nanoparticles to the apical surface. Nanoparticles are located between the basal plasma membrane and the transwell insert (arrows), for (b) the lower area is also magnified in the top left corner ×2. (c) A gold nanoparticle in the intercellular junction of hCMEC/D3 cells (arrow) 3 hours after application of nanoparticles to the apical surface (detailed magnification of the junction with nanoparticle is in the top left corner ×7). The nanoparticles are also seen in the cytosol and vesicles. (d) Detail of nanoparticles located in the cytosol and beneath the basal membrane. (e) Detail of nanoparticles in a vesicle. Scale bars = 500 nm.

The detected gold nanoparticles were counted and sorted into 6 different categories according to their localization: upper membrane, lower membrane, cytosol, vesicles, junctions and nucleus (Table 1). The initial experiments were carried out with primary human brain endothelium (passage-1) or the brain endothelial cell line hCMEC/D3 grown on transwell inserts. The results showed that at time points 3 hrs and 8 hrs, large numbers of nanoparticles were located below the basal plasma membrane (**Fig. 1a, 1b**). These nanoparticles had accumulated in the extracellular matrix between the basal plasma membrane and the transwell insert, as they cannot enter the polyester membrane of the insert, except at the pores. At 1–8 hrs the nanoparticles were also observed in the cytosol but there were very few nanoparticles in vesicles, the nucleus or in cellular junctions (**Fig. 1c** **)**. Higher magnification images confirmed that neither the cytosolic nanoparticles nor the vesicular nanoparticles were enclosed in a phospholipid bilayer, and that the nanoparticles at the basal membrane were extracellular, confirming that they had crossed the cells (**Figs. 1d, 1e**).

The presence of nanoparticles in the cytosol and their virtual absence from cellular junctions suggested that they were directly crossing the cells and were not reaching the basal plasma membrane by the paracellular route. Nanoparticles were seen in vesicles of hCMEC/D3 cells at 22 hrs (data not shown) but at this time, they were in clumps and fewer were located beneath the basal plasma membrane. Hence, in the early stages (3–8 hrs) the nanoparticles appeared to cross the endothelium by non-vesicular transport, but at the last time-point (22 hrs) they were mostly aggregated (>50 nanoparticles per aggregate) and located in vesicles.

### Glucose-coated Gold Nanoparticles Preferentially Cross Brain Endothelia

**Figure 2:**
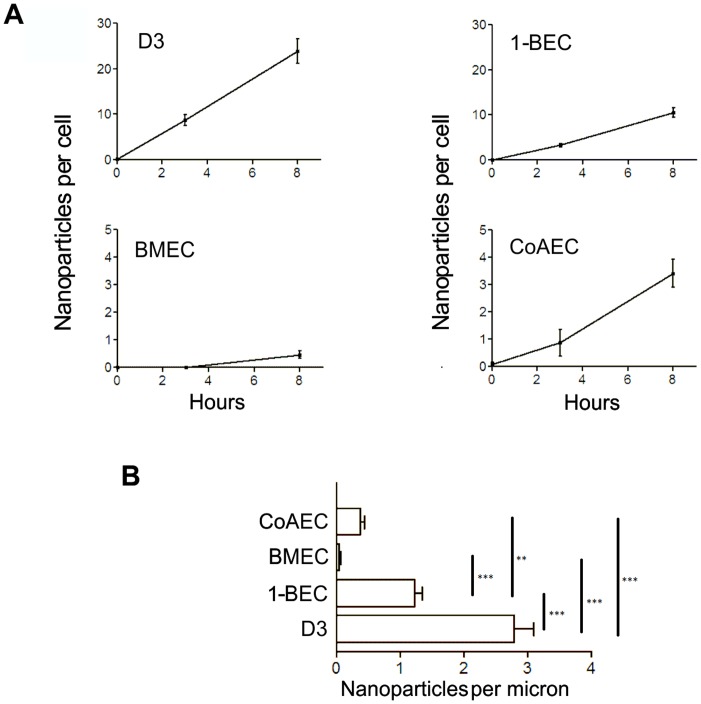
The rate of transfer of glucose-coated gold nanoparticles across brain endothelium compared with non-brain endothelium. (a) Brain endothelial hCMEC/D3 cells and human primary brain endothelium (1-BEC) were compared with a human bone marrow endothelial cell line (BMEC) and human primary coronary artery endothelium (CoAEC). The values show the number of nanoparticles per cell, located between the basal plasma membrane and the transwell insert 8 hrs after application to the apical surface. Values show mean ±SEM from at least 50 different cells, and two separate cultures. Note that the scale of the y-axis is expanded for the two non-brain endothelial cell types. (b) The bar chart shows the number of nanoparticles per micron of the basal membrane after 8 hrs (mean ±SEM) from the four different cell types. Data was analysed by Anova (P<0.05) followed by Tukey’s test to compare each pair of points. **P<0.01, ***P<0.001.

Next, we investigated the rate of transport in endothelia from different tissues; we compared the two sources of brain endothelium (primary brain and hCMEC/D3) with primary coronary artery endothelium and a bone marrow endothelial cell line BMEC (immortalised in a similar way to hCMEC/D3 cells). The transport rate across the brain endothelial cell line hCMEC/D3 and the primary brain endothelium was approximately linear over 8 hours (**Fig. 2a**). Moreover, transport across both brain endothelial cell lines was considerably more efficient than across the two non-brain endothelial cells (**Fig. 2b**).

As an additional comparison, we used a non-endothelial cell type, human fibroblasts, in which the rate of movement of the nanoparticles was measured over 5 hrs with the same experimental setup as above. The rate of transfer to the lower membrane of fibroblasts was <3% of the rate of transfer across the primary brain endothelium.

To estimate the total number of nanoparticles that were cell-associated (inside the cell or at the bottom of the cell between the basal plasma membrane and the membrane of the insert) we counted nanoparticles in 1.5 mm (total length of the set of images) × 85 nm (depth of the section) strips from 2 transwell inserts of hCMEC/D3 cells (surface area = 2.55×10^−10^ m^2^).

We counted more than 18,000 nanoparticles in this area. Therefore, the number of nanoparticles in the entire insert (surface area = 1.13×10^−4^ m^2^ ) is 7.9×10^9^ nanoparticles.

Confluent monolayers of hCMEC/D3 cells on these transwell inserts typically contain 10^5^ cells, therefore the number of nanoparticles per cell is 79,000 nanoparticles per cell.

It should be noted that this is a conservative estimate, since it takes no account of failure to detect some of the nanoparticles by TEM or nanoparticles that have moved down through the pores of the filter.

### Transport of Glucose-coated Gold Nanoparticles is by Passive Uptake

**Figure 3:**
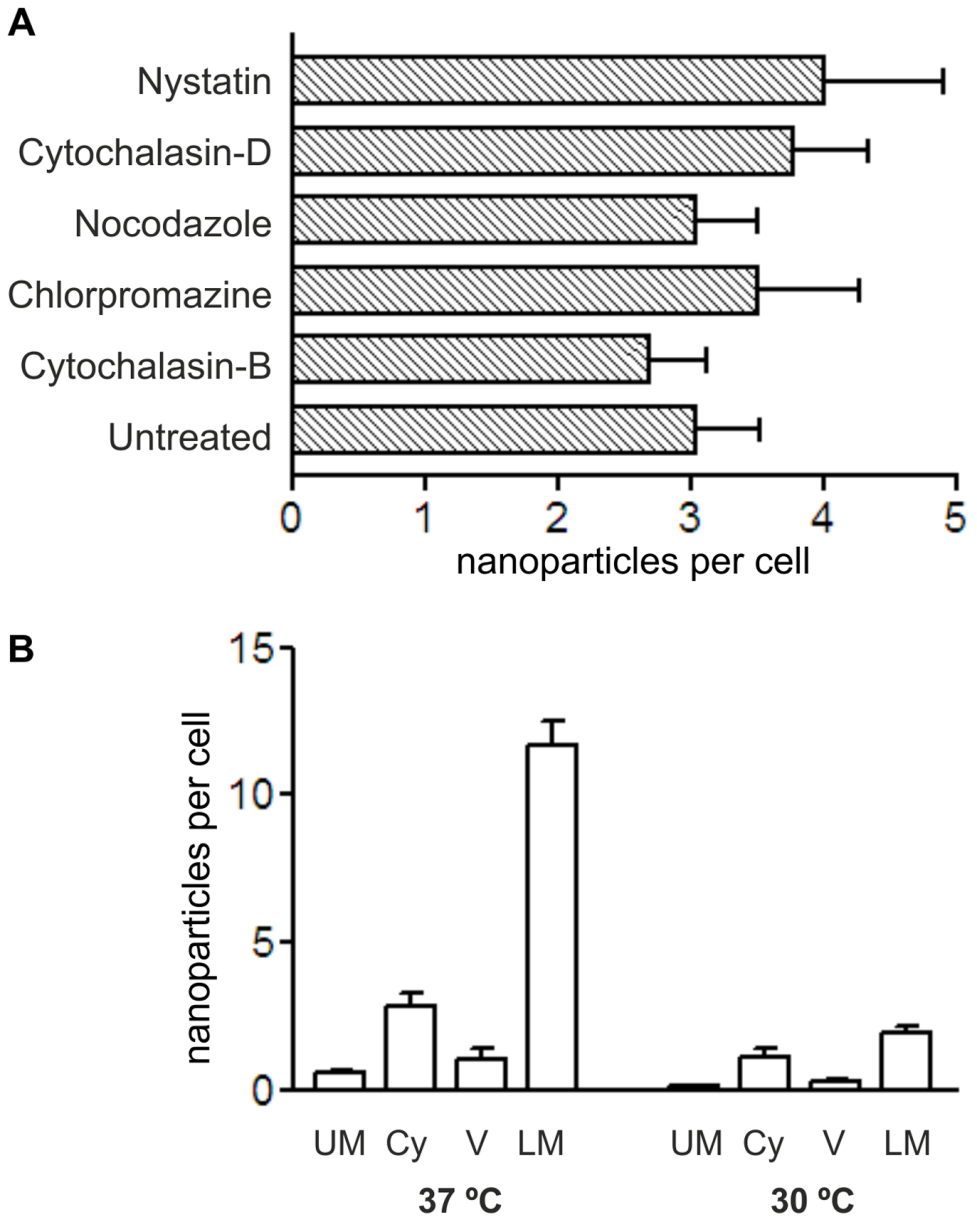
The effect of inhibitors of active cellular transport on the localization of glucose-coated nanoparticles in hCMEC/D3 cells. (a) Cells were treated with 10 µg/ml nystatin, 5 µg/ml cytochalasin-D, 5 µg/ml nocodazole, 10 µg/ml chlorpromazine, 5 µg/ml cytochalasin-B. Data are expressed as the number of nanoparticles located below the basal membrane compared with untreated cells. Values are the mean ± SEM of at least 50 cells. Anova indicates no significant difference between treatments. (b) Localization of nanoparticles in hCMEC/D3 cells at 8 hours after application following incubation at 37°C or 30°C. U.M. = upper (apical) membrane, Cyt. = cytoplasmic, Ves. = vesicular, L.M. = lower (basal) membrane. The values are the mean ± SEM from at least 50 TEM images from a representative experiment. Data was analysed by Anova; there was no significant difference between the control and antibiotic treated samples (P = 0.703).

To further investigate how the nanoparticles were traversing the cell, experiments were carried out for 3 hours using hCMEC/D3 cells in the presence of agents that inhibit endocytosis and/or vesicular transport, namely: chlorpromazine (clathrin-coated vesicles), nocodazole (microtubules), cytochalasin-D (microfilaments) and nystatin (caveolae and lipid rafts) [34]–[37]. If the nanoparticles are transported by a particular vesicular system, then the treatment should block transcytosis. The results showed that at 3 hrs none of these treatments reduced the rate of nanoparticle transfer (**Fig. 3a**). If vesicular transport is excluded, one remaining mechanism for the transfer of nanoparticles across the cells is by passive diffusion across the apical plasma membrane, the cytosol and the basal membrane. Since the plasma membrane limits free diffusion of hydrophilic molecules, we reasoned that changing membrane fluidity (viscosity) would affect the rate of transfer. (Membrane fluidity of mammalian cells is highly temperature-dependent between 37°C and 30°C, while the rate of diffusion is only marginally reduced). We found that reducing the incubation temperature to 30°C reduced the number of nanoparticles in the cytosol by 50% and the transfer rate to the basal membrane by >80% (**Fig. 3b**).

### Transfer Rate Depends on the Coating of the Nanoparticle

We investigated the role of the ligand-coating on the rate of transport. Initially, glucose-coated gold nanoparticles were selected in this study because the glucose transporter, Glut-1 is expressed on brain endothelium and astrocytes [38], [39]. However, cytochalasin-B which inhibits this transporter, had no effect on the rate of transport of these nanoparticles (**Fig. 3a**).

**Figure 4:**
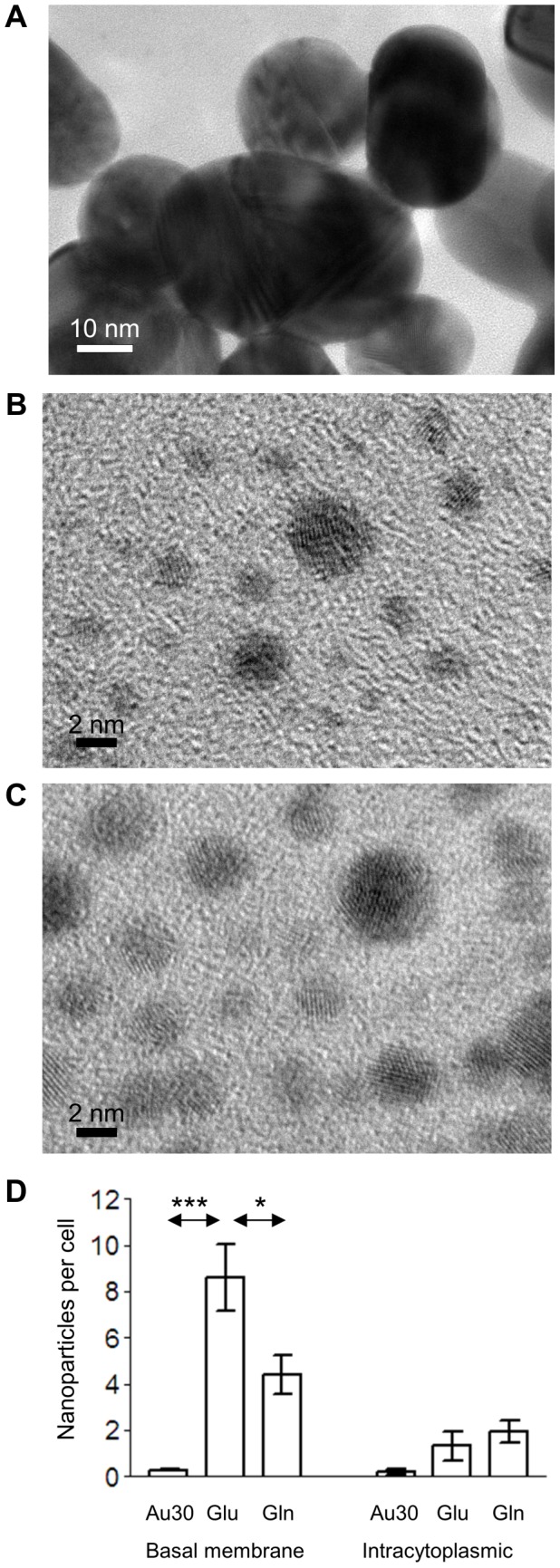
The rate of transport of different gold nanoparticles. TEM of (a) 30 nm colloidal gold (Au30), (b) 4 nm glucose-coated nanoparticles (Glu) and (c) 4 nm glutathione-coated nanoparticles (Gln). (d) Rate of transport of the nanoparticles into and across hCMEC/D3 cells 22 hours after application. Values represent mean ± SEM of the number of nanoparticles located beneath the basal plasma membrane or in the cytosol, based on at least 50 TEM images. Data were analysed by Anova (P<0.01 for the basal membrane data), followed by two-tailed t-tests. *P<0.05, ***P<0.001.

We then compared glucose-coated nanoparticles with glutathione-coated 4 nm nanoparticles to investigate further the importance of coating and 30 nm colloidal gold nanoparticles to investigate the size dependence on the transport (**Fig. 4**). Glucose-coated particles transferred more efficiently than glutathione-coated nanoparticles and both 4 nm coated nanoparticles were far more effective than the 30 nm colloidal gold nanoparticles. This result indicates that the coating of the nanoparticle affects the effectiveness of the transfer, even if the nanoparticle is not using a cellular ligand-specific transport system.

### Glucose-coated Gold Nanoparticles Travel through a 3D Co-culture Model of the Blood-brain Barrier

The ultimate aim of the project was to determine whether the nanoparticles could act as a carrier across the blood-brain barrier and target glial cells. In the initial experiments we had noted that the nanoparticles accumulated between the basal plasma membrane of the endothelium and the transwell insert. Moreover, the nanoparticles were also seen moving through the pores (400 nm in diameter) of the polyester membrane of the transwell insert (the nanoparticles cannot enter the membrane itself), which indicated that they could be released by the endothelium and potentially enter the interstitial spaces.

**Figure 5:**
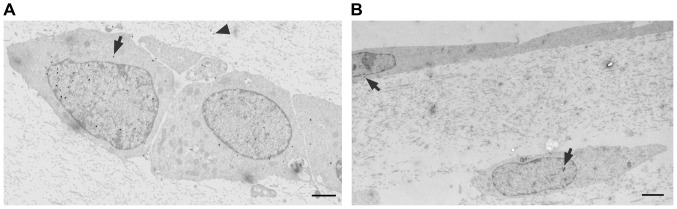
Presence of glucose-coated gold nanoparticles in primary human astrocytes and/or brain endothelial cells hCMEC/D3 in 3D collagen gels. (a) Astrocyte culture 8 hours after application of glucose-coated gold nanoparticles to the gel surface. Nanoparticles are visible both in the gel matrix and the astrocytes (arrows). (b) Co-culture of astrocytes and hCMEC/D3 cells 8 hours after application of glucose-coated gold nanoparticles to the endothelial surface. Nanoparticles are detected both in the endothelium and the astrocyte (arrows). Small tears in the gel matrix are sometimes produced during the sectioning due to the presence of silver-enhanced gold nanoparticles. Scale bars = 500 nm.

To assess the potential of the nanoparticles to target glial cells, we used a novel co-culture system in which human astrocytes were cultured in a 3-dimensional collagen gel, overlaid with a monolayer of human brain endothelium (hCMEC/D3). Preliminary experiments using TEM confirmed that the nanoparticles could pass freely through the gel matrix and enter the astrocytes (**Fig. 5a**). The nanoparticles were then applied to the endothelium in co-culture and the rate of accumulation in astrocytes was measured over 1–8 hrs. Observations were made from a sufficient number of images, to include at least 50 astrocytes containing nanoparticles (**Fig. 5b**). Over the 8 hr time course there was a progressive increase in the percentage of astrocytes with detectable nanoparticles (Table 2).

**Table 2:** Accumulation of glucose-coated gold nanoparticles in human primary astrocytes in co-cultures.

Time^a^	Cells^b^	% positivecells^c^	Distance^d^	Particles/cell^e^
1 hour	411	7.4±2.0	10.6±1.6[max 28]	3.53±0.41
3 hours	308	15.9±1.0	16.7±2.6[max 37]	4.16±0.46
8 hours	240	19.5±0.6	15.5±1.4[max 43]	3.75±1.15

a Time after application of nanoparticles to the apical surface of the brain endothelium (hCMEC/D3).

b Total number of astrocytes observed.

c Percentage of astrocytes with intracellular nanoparticles, mean ± SEM.

d The distance of each astrocyte containing nanoparticles from the basal surface of the endothelium in µm, mean ±SEM. Figures in brackets indicate the maximum distance observed.

e Number of nanoparticles observed in cells containing nanoparticles, mean ±SEM.

In order to check that the nanoparticles were not diffusing into the collagen gel around the edge of the culture (i.e. where the 3D collagen culture meets the wall of the transwell insert), we compared the numbers of nanoparticles at the edge and middle of the transwell inserts. If particles were diffusing from the edge we would expect higher numbers at the edge of the transwell inserts. In practice, the density of nanoparticles was higher in both astrocytes and endothelium in the middle of the cultures, although the difference was not statistically significant for either cell type (Table 3).

**Table 3:** Location of glucose-coated gold nanoparticles in co-cultures.

Cell type	Edge of gel^a^	Middle of gel^a^	P-value(t-test)
Brain endothelium	57.1±14.7	92.4±23.5	0.25
Astrocytes	16.5±1.6	31.8±9.5	0.16

a Nanoparticles per mm located in sections 85 nm deep at 8 hrs, in brain endothelium or astrocytes (mean ± SEM, n = 3 or 4). Data-points were obtained by counting all nanoparticles in strips of 1–2 mm.

Within the 3D collagen gel, astrocytes containing nanoparticles are positioned at different depths from the endothelial monolayer and it was possible to detect the spread of nanoparticles to deeper astrocytes over 1–8 hrs, although the numbers of particles detected per cell was similar at all times (Table 2). As the number of astrocytes with detectable nanoparticles reaches a plateau at 3–8 hr, it suggests that the nanoparticles can pass through astrocytes as well as endothelial cells, and hence they are not accumulating in either cell type during this period. At 1 hour, the median distance of the nanoparticles in astrocytes from the endothelium was 10.6 µm and the maximum distance was 28 µm, suggesting the nanoparticles can permeate the gel moving on average at ∼10 µm per hour.

We then estimated the number of nanoparticles per astrocyte. The sections produced for electron microscopy were 85 nm thick, and all nanoparticles within the astrocytes in these sections were counted. We observed on average 3.75 nanoparticles/cell at the 8 hour time point (Table 2). For a single astrocyte, up to 85 µm in diameter, only 0.1% of the total nanoparticles are visible in the 85 nm section and we infer that each astrocyte could therefore contain several hundred nanoparticles.

### Cytotoxicity of Coated Gold Nanoparticles

**Figure 6:**
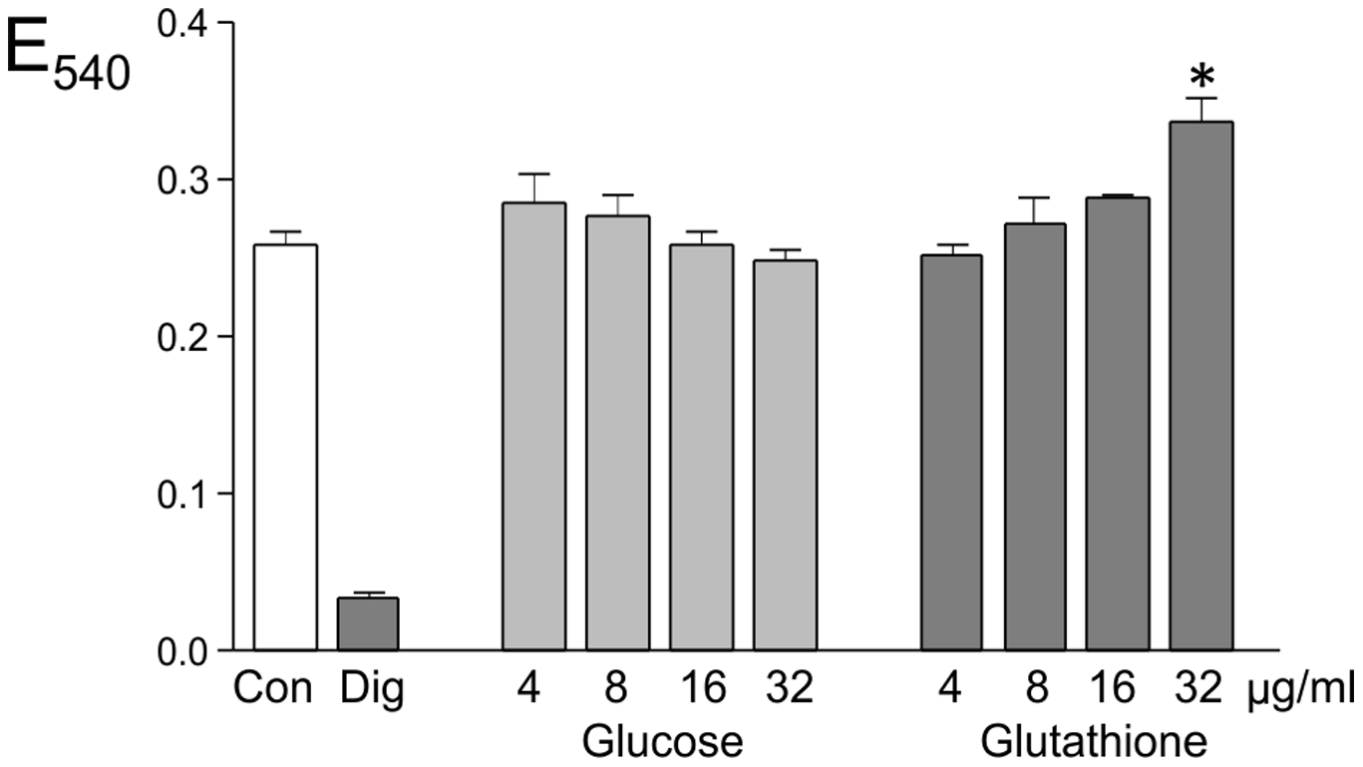
Viability of hCMEC/D3 cells treated with gold nanoparticles. Confluent monolayers of hCMEC/D3 cells were treated for 24 hr with different levels of either glucose-coated or glutathione-coated gold nanoparticles and viability was assessed by MTT assay. Values are mean and SEM of quadruplicate determinations (n = 4). The values were analysed by Anova (p<0.001) followed by Dunnet’s multiple comparison test, comparing each nanoparticle treatment with the untreated cells (Con). Only one treatment was significantly different from the control (*P<0.001). Digitonin-treated cells (Dig) were a positive control for cell death.

To assess potential toxicity of the nanoparticles we performed an MTT viability assay on hCMEC/D3 cells exposed to 4, 8, 16 or 32 µg/ml nanoparticles for 24 hrs (**Fig. 6**). There was no reduction in the viability of the cells at any of the doses tested. In one experiment, there was a significant increase in the absorbance (optical density) of the cells treated with the highest dose of glutathione-coated nanoparticles, which may be due to direct absorbance by cell-associated gold nanoparticles. However, the increase was not significant in 2 further repeats of the assay.

## Discussion

Targeted delivery of drugs to cells of the CNS is a major obstacle in the treatment of many diseases. Gold nanoparticles have considerable potential as carriers of therapeutic agents across the blood-brain barrier. This study shows that glucose-coated gold nanoparticles are potential carriers for therapeutic agents into the brain. We found that these nanoparticles are localized in the cytosol rather than in endosomes, decreasing the risk for potential degradation of the cargo. Moreover, they are preferentially taken up by brain-endothelium compared to non-brain endothelia and have low cytotoxicity.

Gold nanoparticles are not immunogenic and smaller nanoparticles (3–5 nm) are not cytotoxic except at high doses [40]–[42]. The glucose-coated gold nanoparticles used here caused no reduction in viability of the endothelium following 24 hours treatment. The study also demonstrated that the glucose-coated gold nanoparticles can selectively cross human brain endothelium *in vitro* and localise in astrocytes.

The 2D and 3D culture systems used in this study allowed quantitation of the rate of transfer across brain endothelium and analysis of the cellular mechanisms. The use of human cells is also important since there are differences in the composition of the blood-brain barrier between species. However, by comparison with the situation *in vivo*, the barrier *in vitro* is less tight for ions and smaller molecules [28]. As we were using static cultures, we considered the possibility that sedimentation of the particles could produce the results seen here. However, in the case of gold nanoparticles less than 15 nm, sedimentation is negligible and should not have an effect on the transport mechanism [43]. We also considered the possibility that the nanoparticles could reach the base of the endothelium by diffusion around the edge of the culture wells. However, diffusion around the edge of the cultures was excluded because there was no significant difference between the numbers of nanoparticles at the centre and at the edge of the cultures. Thus the culture systems appear to be suitable for assessing trans-endothelial movement and subsequent localisation of nanoparticles of this size (27 kDa).

Originally, we investigated glucose-coated nanoparticles due to their possible binding to the glucose transporter Glut-1, present on brain endothelium and astrocytes [38], [39]. The finding that these nanoparticles were selectively transported by brain endothelium, by comparison with non-brain endothelium, initially supported the view that the transfer was cell type specific and ligand-dependent. However, the transfer was not blocked by antibiotics that interfere with endocytosis or cytochalasin-B which blocks glucose uptake. These results imply that transcytosis (which is normally low in brain endothelium) and the glucose transporter are not responsible for the transfer of the glucose-coated nanoparticles. Possibly, the physical configuration of the glucose, in tightly-packed rings around the 2 nm gold core, means that it cannot engage the Glut-1 transporter effectively [44]. An alternative explanation for the brain-selectivity is that transfer depends on other tissue-specific properties of endothelial cells. In this respect, the surface glycocalyx of brain endothelium is quite different from endothelium in other tissues, with a very high negative charge [24]. Other studies have implied that the surface charge of gold nanoparticles affects their ability to penetrate the plasma membrane; cationic nanoparticles are taken up more efficiently than anionic nanoparticles [23]. If the charge on the endothelial apical plasma membrane is important in controlling the rate of transfer, then one would predict that nanoparticles coated with glucose (uncharged) would be transferred more effectively than those coated with glutathione, which has a negative charge. This is indeed the case.

Other studies have shown that the type of coating can affect the uptake of this class of nanoparticle, and critically determine whether they enter endosomes or directly penetrate the plasma membrane [22]. Since the nanoparticles were seen primarily in the cytosol and in much smaller numbers in vesicles, the simplest explanation is that the nanoparticles travel across the endothelium itself mainly via the cytosol, which means that they must also cross the apical and basal plasma membranes. Reducing the temperature to 30°C reduced the number of particles in the cytosol by 50% and the rate of transfer across the cell by more than 80%. This result is as expected for nanoparticles crossing the apical and basal plasma membranes, assuming that membrane fluidity is an important determinant of the transfer rate. The reduced rate of transfer cannot be explained by a reduction in the diffusion constant for the nanoparticles, which is only marginally reduced between 37°C and 30°C. However, we cannot exclude the possibility that some other cellular process, which is highly temperature-dependent, could produce this reduction.

On transwell inserts, the nanoparticles accumulated in the extracellular space between the basal plasma membrane and the polyester membrane of the transwell insert. In the 3D co-cultures, the nanoparticles are free to move away from the endothelium and their distance from the endothelial monolayer increased over 1–3 hrs (Table 2). They then appeared to accumulate in the astrocytes, but this appearance may be because they move more slowly through cells than the gel matrix. The localisation of nanoparticles in co-cultures provided surprising data. It was notable that nanoparticles were rarely seen in the nuclei of the endothelium, but common in the nuclei of astrocytes, either in single cell cultures or co-cultures. Previous work on gold nanoparticles with a structured surface also showed that they were completely excluded from the nucleus [22].

It is possible that changes in the surface coating of the nanoparticles occur during the passage through the endothelium, which means that they subsequently tend to localise to the astrocyte nucleus. One possibility is that the reducing environment of the endothelial cytosol causes release of some of the covalently-bound glucose, resulting in a change in their charge and/or ability to bind protein [33], which affects their ability to move through different membranes, including the nuclear membrane. For example, it has previously been shown that organic thiol ligands can be released from the nanoparticle surface by exchange with cellular glutathione [45]. However, any change in the properties of the nanoparticle in the co-culture did not cause the nanoparticles to aggregate; it is important that the nanoparticles are not trapped in the endothelium if they are to be used to deliver a therapeutic cargo to cells of the CNS.

The number of transferred nanoparticles is also an important consideration. Our calculations suggest that >70,000 nanoparticles crossed each endothelial cell and several hundred accumulated in each astrocyte. They therefore have the potential to carry an effective dose of a toxic agent, a receptor agonist or a gene to the target cells, if the process can be made to occur at a similar level *in vivo*. Nanoparticles of this class are currently undergoing trials in humans for the treatment of a number of diseases. It appears that these smaller nanoparticles are less rapidly removed by the mononuclear phagocyte system than larger ones [10]. Treatment of CNS disease with nanoparticles has not yet been attempted in humans, partly because of problems associated with getting them to cross the blood-brain barrier. Studies in animals have shown that gold nanoparticles can be used for drug-delivery or for imaging in the CNS or for enhancing radiotherapy of brain tumours [8], [9], [46]. Our study shows that 4 nm glucose-coated gold nanoparticles are able to move across the cell through its cytosol, are selective for human brain endothelium and are able to enter the astrocytes. We imply that they have potential as a delivery system for therapeutic agents to cells within the CNS, and we are currently developing the nanoparticles as a gene-delivery system. We are also first to examine the transport system of nanoparticles in co-culture of two different cell types at the same time *in vitro*.

## Supporting Information

Figure S1 **The experimental setup for gold-nanoparticle experiments with transwell inserts.** Each experimental treatment (or control) has been performed in duplicates in a single experiment and three independent experiments were performed.
(TIF)
