# Supplementary figures and images for: Glucose-Coated Gold Nanoparticles Transfer across Human Brain Endothelium and Enter Astrocytes In Vitro
Source: PLoS One. 2013 Dec 5;8(12):e81043. doi: 10.1371/journal.pone.0081043 (PMC3855187; doi:10.1371/journal.pone.0081043)

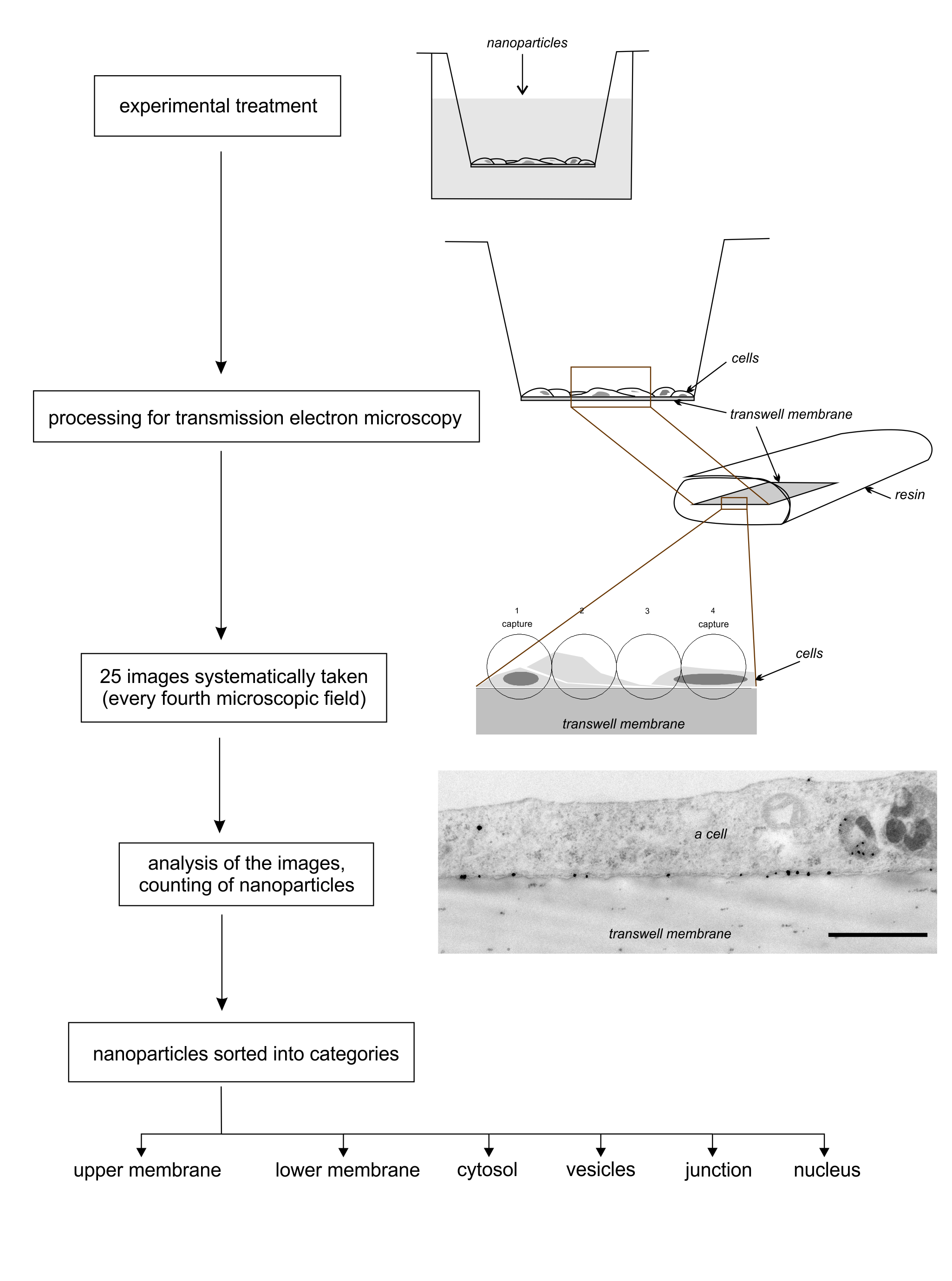

Supplement: Figure S1 — The experimental setup for gold-nanoparticle experiments with transwell inserts. Each experimental treatment (or control) has been performed in duplicates in a single experiment and three independent experiments were performed. (TIF) [file pone.0081043.s001.tif]
